# Metabolic syndrome among residents of Mizan-Aman town, South West Ethiopia, 2017: A cross sectional study

**DOI:** 10.1371/journal.pone.0210969

**Published:** 2019-01-31

**Authors:** Sitotaw Kerie, Melak Menberu, Mathewos Geneto

**Affiliations:** 1 Department of Nursing, College of Medicine and Health Sciences, Bahir Dar University, Bahir Dar, Ethiopia; 2 Department of psychiatry, College of Medicine and Health Sciences, Bahir Dar University, Bahir Dar, Ethiopia; 3 Department of Medicine, College of Medicine and Health Sciences, Wachemo University, Hossana, Ethiopia; University of Maiduguri College of Medical Sciences, NIGERIA

## Abstract

**Introduction:**

Globally, it is estimated that around 20–25% adult population has metabolic syndrome. Individuals who have metabolic syndrome are up to five times more susceptible for chronic diseases than those who have no metabolic syndrome. In Ethiopia there is no sufficient information regarding the magnitude and factors of metabolic syndrome. The aim of this study is to assess prevalence and associated factors of metabolic syndrome among residents of Mizan-Aman town, South West, Ethiopia.

**Methods:**

The community based cross-sectional study was held at Mizan-Aman town residents. Systematic random sampling was employed to select each household and lottery method was used to select one individual from the household. Data were cleaned, coded and entered by EPI-INFO version 3.5.4 and were transported to SPSS version 20 for further analysis. To indicate the strength of association, odds ratios (OR) and 95% confidence intervals (95% CI) were used.

**Results:**

In this study from a total of 558 respondents 534 were completed the interview correctly, which gives a response rate of 95.7%. The overall prevalence of metabolic syndrome was 9.6%. Multivariate logistic regression revealed that physical inactivity [AOR = 2.61, 95% CI (1.22, 5.58)], age from 18 to 28 years [AOR = 0.36, 95% CI (0.14, 0.90)], being male [AOR = 0.46, 95% CI (0.22, 0.96)] and educational status with cannot write and read [AOR = 0.15, 95% CI (0.04,0.53)], from grade 1 to 8 [AOR = 0. 17, (0.11,0.55)], from grade 9 to12 [AOR = 0.11, (0.03, 0.38)] and from diploma to degree [AOR = 0. 13, (0.01, 0.36)] were significantly associated with metabolic syndrome.

**Conclusion:**

The prevalence of metabolic syndrome in this study was found to be high. Age, physical activity, educational status and sex were significantly associated with metabolic syndrome. Physical activity was found to be the means of metabolic syndrome prevention.

## Introduction

Metabolic syndrome is the constellation of metabolic abnormalities which includes central obesity, decreased high density lipoprotein cholesterol, elevated triglycerides, elevated blood pressures and hyperglycemia [1]. Globally, it is estimated that around 20–25% adult population has metabolic syndrome [2]. Metabolic syndrome increases the magnitudes of cardiovascular disorder and stroke by three to ten times and diabetic mellitus by ten times [3]. Among all diabetic type two ill individuals, 80% also had metabolic syndrome [4]. It is thought to be a driver of the modern day epidemics of diabetes and cardiovascular disorder and has become a major public health challenge around the world [1].

Studies conducted on prevalence rates of metabolic syndrome produce various results. For instance, it ranges from 8% in India to 24% in USA among men and from 7% in France to 43% in Iran among women [1]. Metabolic syndrome becomes increasing in the developing world due to lifestyle change resulting from industrialization and migration of rural to urban by decreasing levels of physical activity and intake of energy [5].

In Africa the prevalence of metabolic syndrome ranges from 17% to 25% [6]. Metabolic syndrome is not only increasing the risk of developing non-communicable diseases; but also increases the cost of treatment for non-communicable diseases. It will increase the economic burden of hypertension and other non-communicable diseases by 59% to 179% by 2020 [7]. Even though the metabolic syndrome brings such high diseases and economic burden, we can decrease and prevent it by simple lifestyle modification like weight reduction and using anti-athereogenic diet [7].

The primary means to decrease the burden and consequence of non-communicable diseases like DM and CVD is the identification and prevention of common risk factors. Metabolic syndrome is the most known and prominent risk factor of non- communicable diseases.

Despite; crucially of knowing magnitude and associated factors of metabolic syndrome for prevention of non-communicable diseases, there is currently a gap in the literature, as no studies of metabolic syndrome have previously been conducted in Ethiopia in the general community. Thus, this study will provide evidence regarding the magnitude and factors of metabolic syndrome in the general community of Ethiopia.

## Materials and methods

### Study design and period

A community based cross-sectional study was conducted on 534 participants in Mizan-Aman town, located in South West Ethiopia. Mizan-Aman is located 581 km from Addis Ababa. There are 1 health center, 2 health posts, 11 medium private clinics, 3 rural private clinics, and one teaching referral hospital in Mizan-Aman town. The data were collected from May, 1 to July 30, 2017 for three consecutive months.

### Sample size and procedure

The number of samples required for this study was calculated for each specific objective by considering double population proportion formula by using Epi-Info version-7 for associated factors and the single population proportion formula was employed for the dependent variable. For single population proportion formula, we consider the following assumptions (Table 1):

$$n^{i}=\left(\left([Z\alpha /2\right)]↑2\right)p\left(1-p\right))/(d↑2)\;\mathrm{Where}$$

➢ n^*i*^ = initial sample size required for the study
➢ Z = standard normal distribution (Z = 1.96) with confidence interval of 95% and
➢ P = 18% [8]. Therefore, p is taken as 18% to calculate the sample size.
➢ d = tolerable margin of error (d) = 0.05

$$n^{i}=\left(\left([Z\alpha /2\right)]↑2\right)p\left(1-p\right))/(d↑2)=226.8=227$$

**Table 1:** Sample size calculation by using different variables [9].

Sample size by sex	Sample size by abdominal obesity	Sample size by dependent variable
n = 338 with p = 24%	n = 288with p = 38.9%	n = 227 with p = 18%

We took maximum sample size (n = 338). Since it is multi-stage this sample size was multiplied by the design effect (n = 507) and by adding 10% non-response rate our final sample size is 558.

Finally, proportional sample size was taken from each two sub cities as per the total population.

Systematic random sampling was employed to select each household and lottery method was used to select one individual from the household.

### Data collection procedure

The data collection has three components: first there were questionnaires to collect socio-demographic and other independent variables, second, simple physical anthropometric measurement and lastly bio-chemical measurement. Physical anthropometric measurement and blood sample collection were carried out by trained nurses and laboratory technicians. Blood pressure was done by using digital measuring device. Then three BP measurements were taken after participants sitting for at least five minutes. Waist circumference was taken at the midpoint between the lower margins of lowest palpable rib and the top of the iliac crest (hip bone). Five ml blood sample was taken from each participant before breakfast by employing infection prevention procedure. Fasting glucose level (FGL) was done by using Gluco-meter and serum triglyceride (TG), total cholesterol (TC), HDL, were measured at Jimma university specialized hospital laboratory department.

### Measurements

Metabolic syndrome was screened from the community by using ATP III. ATP III is standardized tool which is recommended by the international diabetes federation to screen metabolic syndrome in the community [8]. Stress was measured by using perceived stress scale. It is developed in 1983 and it has 10 items with a total score of 40 [10]. Physical activity was also measured by using international physical activity questionnaire. It has 7 items which assess health enhancing physical activity, minimal activity and inactivity [11].

### Data quality control

The questionnaire was translated to Amharic and translated back to English. Four days Training was given for data collectors. Pre-test done on (5%) of our sample size in Mizan-Aman town residents who were not part of the study participant. Based on the finding of the pre-test, the questioner was revised. The participants were informed about confidentiality of their information. The data collectors were supervised daily and the filled questionnaires were checked daily by the principal investigators for completeness and errors were identified and corrected.

### Statistical analysis

The collected data were entered and analyzed using SPSS version 20. Descriptive statistics (frequency, mean, median, standard deviation and Percentage) were used to describe socio-demographic characteristics of the study population. Then bivariate logistic regression techniques were done to see the crude association between the independent variables and the Dependent variable. Result from bivariate analysis of p<0. 2 were moved to multivariate analysis to control the effects of confounding and to identify predictors of metabolic syndrome.

A P value of < 0.05 was used as the criterion for statistical significance and OR with 95% confidence interval was used to indicate the strength of association.

### Ethical consideration

The ethical approval letter was obtained from the Institutional Review Board (IRB) of Mizan-Tepi University. Permission letter was taken from town administration. Name and address of the participants were not taken, and participants were informed about the aim of the study, the advantages of the study, and their rights even to stop in the middle of the procedure. Participants also were informed about absent of direct benefits from the study.

However, their participation in this study is very important for achievement of the study and for paving the way for development of the intervention project to prevent metabolic syndrome in Mizan-Aman town. No risk will occur on them because of their participation in this study. All results getting from them; will be kept confidential using password and there will be no need of recording their identities. Finally, written informed consent was taken before data collection.

## Result

### Socio demographic characteristics of respondents

From a total of 558 respondents 534 participants were included in the study, which makes the response rate of 95.7%. Among the respondents, 278 (52.1%) were in the age range of 18–28 years with mean age of 30.91 years with standard deviation of +10.9, more than half of participants 271 (50.7%) were male, 305 (57.1%) were Benchi, 336 (62.9%) were currently married, 195 (36.5%) had educational levels of from grade 1 to grade 8, majority 381 (71.3%) were protestant religion followers **(Table 2).**

**Table 2:** Socio demographic characteristics of participants of Mizan-Aman town, south west Ethiopia, 2017 (n = 534).

Variable	Frequency	(Percent)
Sex		
Male	271	50.7
Female	263	49.3
Age in year		
18–28	278	52.1
29–39	139	26.0
≥40	117	21.9
Marital status		
Single	156	29.2
Married	336	62.9
Other	42	7.9
Educational status		
Cannot read and write	90	16.8
From grade one to eight	195	36.5
From grade 9 to 12	160	30
Diploma Degree and above	66 23	12.4 4.3
Occupational status		
Government employee	124	23.2
Self-employee	172	32.2
Farmer	143	26.8
Jobless	95	17.8

### Life style related characteristics of respondents

Among all respondents only 203 (38%) are doing adequate exercise, the majority of them had intermediate stress 472 (88.4%) and 67 (12.5%) are currently alcohol users **(Table 3).**

**Table 3:** Life style related characteristic of respondents of Mizan-Aman town, south west Ethiopia 2017. (n = 534).

Variable	Frequency	Percent (%)
physical activity		
Yes	203	38
No	331	62
Current alcohol users		
Yes	67	12.5
No	467	87.5
Stress level		
No stress	45	8.4
Intermediate stress	472	88.4
High level stress	17	3.2

### Laboratory and physical examination findings of respondents

There are three laboratory findings (blood glucose level, HDL, TG) and two physical examination findings (blood pressure and waist circumference). More than half of participants had reduced levels of high density lipoprotein 272 (50.9%) while, 122 (22.8%) of them had hyper-triglyceride, 90 (16.9%) had hypertension and 38 (7.1%) of them had elevated fast glucose level. More than one quarter of participants had an abnormal waist circumference (**Table 4**).

**Table 4:** Distribution of respondents by their waist circumference, HDL, blood pressure, level of TG and blood glucose.

Variable	Frequency	Percent (%)
Abnormal waist circumference		
Yes	140	26.22
No	394	73.78
Reduced HDL		
Yes	272	50.9
No	262	49.06
High blood pressure		
Yes	90	16.9
No	444	83.1
High blood glucose		
Yes	38	7.1
No	496	92.9
High TG		
Yes	122	22.8
No	412	77.2

### Prevalence of metabolic syndrome

Among 534 participants 51 (9.6%) of them had metabolic syndrome.

### Factors associated with metabolic syndrome

In bivariate analysis sex, educational status, age, current khat chewing, current alcohol drinking and exercise had P value < 0.25 and included in multiple logistic regression analysis.

During multiple logistic regressions; lack of adequate exercise [AOR = 2.61, 95% CI (1.22, 5.58)], age from 18 to 28 years [AOR = 0.36, 95% CI (0.14, 0.90)], being male [AOR = 0.46, 95% CI (0.22, 0.96)] and educational status with cannot write and read [AOR = 0.15, 95% CI (0.04,0.53)], from grade 1 to 8 [AOR = 0.17, (0.11,0.55)], from grade 9 to12 [AOR = 0.11, (0.03, 0.38)] and from diploma to degree [AOR = 0.13, (0.01, 0.36)] were significantly associated with metabolic syndrome **(Table 5).**

**Table 5:** Logistic regression of associated factors and metabolic syndrome among residents of Mizan-Aman town, south west Ethiopia, 2017 (n = 534).

Variable	Metabolic syndrome No(n)	Yes(n)	COR (95%CI)	AOR(95%CI)
Sex = male	253	18	0.56(0.27,0.91)	0.46(0.22,0.96)
= female	230	33	1	1
Educational status				
Cannot write & read	76	14	0.35(0.12,0.97)	*0.15(0.04,0.53)
From grade1-8	176	19	0.20(0.08,0.54)	*0.17(0.11,0.55)
From grade 9–12	152	8	0.19(0.03,0.30)	*0.11(0.03,0.38)
Diploma	64	2	0.19(0.01,0.31)	*0.13(0.01,0.36)
Degree and above	15	8	1	1
Age = 18–28 year	268	10	0.22(0.17,0.55)	0.36(0.14,0.90)
= 29–39 year	115	24	1.23(0.62,2.42)	1.67(0.79,3.52)
= ≥ 40 year	100	17	1	1
khat chewing = yes	7	3	4.25(1.06,16.97)	1.28(0.12,13.36)
= no	476	48	1	1
Alcohol drinking = yes	57	10	1.82(0.87,3.84)	1.17(0.45,3.04)
= no	426	41	1	1
Adequate exercise = yes	192	11	1	1
= no	291	40	2.49(1.20,4.79)	2.61(1.22,5.58)

NB: written in thick indicates significant association

*(star sign) = p-value<0.01

## Discussion

Among all participants, 9.6% with 95% CI (7.01, 12.19) had metabolic syndrome. Physical activity, sex, educational status and age of participant were significantly associated with metabolic syndrome. In this study magnitude of metabolic syndrome is in line with a study done in Japan which was 7.8% [12]. But, it is found to be low when it is compared with studies done in the USA (29.4%), Nepal (22.5%), Canada (52.5%), Ghana (15%) and Nigeria (18%) [13–17]. The possible reason for this discrepancy may be the difference in sample size, study period, and study setting. For example, in Ghana the sample size was 176 while in this study the sample size was 534. Moreover, in the USA, Canada and Nepal abusing alcohol is more common than in Ethiopia, which is the most known factor that accelerate the chance to develop metabolic syndrome. Magnitude of metabolic syndrome in this study is higher than when it is compared with the finding of a study conducted at Cameron (less than 2%) [18]. The possible reason for this discrepancy may be the above study includes both urban and rural residents, whereas our study includes only urban residents. Living in urban increases risk of developing metabolic syndrome by increasing sedentary life style.

Physical activity was significantly associated with metabolic syndrome. Participants who have physical inactivity are 2.61 times more likely to develop metabolic syndrome than those who had adequate physical activity. Having regular physical activity is one mechanism of decreasing metabolic syndrome components like obesity, type II DM and hypertension. Even, after we develop metabolic syndrome regular physical activity is one means of preventing complication of metabolic syndrome [19]. Previous Studies support that adequate physical activity is the non-pharmacological treatment of genetic obesity and type II DM and it is also non-pharmacological prophylaxis of metabolic syndrome. Since exercise has anti-inflammatory effect, it is the best means of prevention of chronic low-grade inflammation which is caused by metabolic syndrome [20]. Some studies prove that regular physical activity is the cornerstones of prevention of metabolic syndrome and risk factors of cardiovascular disorder and diabetics Miletus. The individual with physical activities are 53% less risk for metabolic syndrome than those who didn’t do physical activities [21].

Age of participants was significantly associated with metabolic syndrome. Participants whose age are with a range of 18 to 28 years are 64% less likely to develop metabolic syndrome than those who has age is greater than or equal to 40 years. Age and risk of developing metabolic syndrome are directly proportional. When age increases risk of developing metabolic syndrome also increases [22]. Mostly metabolic syndrome starts from middle age [23]. Among men’ and women whose age is older than 65 years more than 3l.5% and 59.8% of them full fills the criteria of metabolic syndrome, respectively. Some studies also prove that the prevalence of metabolic syndrome is higher among female than male [24].

Sex was also significantly associated with metabolic syndrome. Males are 54% less likely to develop metabolic syndrome when compared to females. The possible reason may be Ethiopian cultures does not encourage females to do physical activity which makes them more vulnerable to metabolic syndrome than males.

The other variable which was significantly associated with metabolic syndrome was educational status of participants. Individuals with educational status of below degree are up to 89% less likely to develop metabolic syndrome than those with educational status of degree and above.

The possible reason may be, those individuals who have higher educational status are mostly employed. This employment, in turn, forces them to be in office. As known office work is not allowing physical activity rather than a sedentary life. The sedentary life is the most prominent and independent factors of metabolic syndrome [25].

## Conclusion

The prevalence of metabolic syndrome in this study was found to be high. Age, physical activity, educational status and sex were significantly associated with metabolic syndrome. Physical activity was found to be the means of metabolic syndrome prevention.

## Supporting information

S1 Data Supplementary file (spss data). (SAV)

## Abbreviations

ATP III: Adult Treatment Panel
HDL: High Density Lipoprotein
LDL: Low Density Lipoprotein
FGL: Fasting Glucose level
TG: Triglycerides
WC: Waist Circumference
U.S.A: United States of America
WHO: World Health Organization
METS: Metabolic Syndrome
DM: Diabetic Miletus
IPAQ: International physical activity questionnaire
